# Multiple non-collinear TF-map alignments of promoter regions

**DOI:** 10.1186/1471-2105-8-138

**Published:** 2007-04-24

**Authors:** Enrique Blanco, Roderic Guigó, Xavier Messeguer

**Affiliations:** 1 Grup d'Algorísmica i Genètica. Departament de Llenguatges i Sistemes Informàtics. Universitat Politècnica de Catalunya. C/Jordi Girona, 1–3, 08034 Barcelona, Catalonia, Spain; 2 Bioinformatics and Genomics Program, Centre de Regulació Genòmica.Pg. Marítim de la Barceloneta 37–49, 08003 Barcelona, Catalonia, Spain

## Abstract

**Background:**

The analysis of the promoter sequence of genes with similar expression patterns is a basic tool to annotate common regulatory elements. Multiple sequence alignments are on the basis of most comparative approaches. The characterization of regulatory regions from co-expressed genes at the sequence level, however, does not yield satisfactory results in many occasions as promoter regions of genes sharing similar expression programs often do not show nucleotide sequence conservation.

**Results:**

In a recent approach to circumvent this limitation, we proposed to align the maps of predicted transcription factors (referred as TF-maps) instead of the nucleotide sequence of two related promoters, taking into account the label of the corresponding factor and the position in the primary sequence. We have now extended the basic algorithm to permit multiple promoter comparisons using the progressive alignment paradigm. In addition, non-collinear conservation blocks might now be identified in the resulting alignments. We have optimized the parameters of the algorithm in a small, but well-characterized collection of human-mouse-chicken-zebrafish orthologous gene promoters.

**Conclusion:**

Results in this dataset indicate that TF-map alignments are able to detect high-level regulatory conservation at the promoter and the 3'UTR gene regions, which cannot be detected by the typical sequence alignments. Three particular examples are introduced here to illustrate the power of the multiple TF-map alignments to characterize conserved regulatory elements in absence of sequence similarity. We consider this kind of approach can be extremely useful in the future to annotate potential transcription factor binding sites on sets of co-regulated genes from high-throughput expression experiments.

## Background

Sequence comparisons are one of the most important computational tools in molecular biology. Sequences are good symbolic representations of biological molecules that encode relevant information about their structure, function and history. From the analysis of functionally related sequences, biologically significant facts can be inferred. For instance, genomic sequence comparisons are performed in order to identify genes or regulatory sites across evolutionarily related genomes, as these functional elements tend to exhibit conservational patterns different from those observed in regions that are not functional.

In attempt to allow for multiple sequence comparisons, the basic dynamic programming recurrences introduced in the 1970s to align efficiently two sequences of *n *symbols in time proportional to the square of the length of the sequences [1,2], can be naturally extended for *k *sequences, with an exponential cost *O*(*n*^*k*^)[3]. As this cost is unaffordable in practice, many heuristics have appeared to provide acceptable solutions with a minor cost. The most popular of them is the progressive alignment [4,5].

This procedure is a greedy algorithm that runs in *O*(*k*^2^*n*^2^) time. In a first step, the progressive alignment performs all of the pairwise alignments to build an evolutionary tree. In a second step, an initial alignment is constructed from the two closest sequences, incorporating then the rest to the profile following the guide tree. Such a procedure does not guarantee to find the optimal solution in mathematical terms. However, the results are generally meaningful from the biological standpoint.

These comparisons at the sequence level have limitations however. Although similar sequences do tend to play similar biological functions, the opposite is not necessarily true. Often similar functions are encoded in higher order sequence elements that are not necessarily conserved at the sequence level. As a result, similar functions are frequently encoded by diverse sequences which are undetectable by conventional sequence alignment methods.

Gene promoter regions are a good example. The information that governs the RNA synthesis is mostly encoded in the gene promoter, a region normally 200 to 2000 nucleotides long upstream of the transcription start site of the gene (TSS). Transcription factors (TFs) bind to sequence specific motifs (the TF binding sites, TFBSs) within the promoters. TFBSs are 5–8 nucleotides long and one promoter region contains on the order of 10 to 50 of them [6]. Such motifs appear to be arranged in specific configurations that define the temporal and spatial transcriptional program of each gene. Genes presenting similar expression patterns are assumed to share similar configurations of TFBSs in their promoters [7,8].

However, TFBSs associated to the same TF show often little sequence conservation. Therefore, promoter regions of genes with similar expression pattern may not be similar at the sequence level.

In a previous work [9], we showed that pairwise alignments between sequences of labels representing TFs binding to sites predicted in promoter regions (TF-maps) could often uncover high-level common regulatory patterns which could not be found by typical nucleotide sequence comparisons.

Here, we present an efficient implementation of the multiple TF-map alignment based in the progressive alignment paradigm. We have introduced some modifications in the pairwise global TF-map alignment algorithm to allow the alignment of TF-map alignments. In addition, we have extended the algorithm to allow for non-collinear alignments, which are rarely considered in conventional dynamic programming algorithms, being only partially identified by combining global and local alignment strategies [10,11]. The ability to predict non-collinear alignments may be particularly relevant in the case of promoter regions, where the linearity of TFBSs configurations can be weakly conserved [12].

The structure of the paper is the following: first, we briefly review the concept of TF-map and provide the formal definition of a multiple TF-map alignment. Then, we introduce the algorithm to compute the optimal pairwise alignment of two alignments. Next, we describe the main algorithm that performs the progressive alignment of multiple TF-maps. Later, we define formally a non-collinear alignment, introducing some modifications in the basic algorithm. Finally, we systematically estimate the optimal parameters of the alignment to distinguish promoters from other gene regions in a set of well characterized human-rodent gene pairs extracted from the ABS database [13] and their corresponding orthologs in chicken and zebrafish. These results are compared to those obtained by conventional sequence alignment methods. Three particular examples are presented in which multiple TF-map alignments characterize conserved regulatory elements that are otherwise imperceptible in sequence-level comparisons.

### TF-maps

Let ∑_*DNA *_be the alphabet of four nucleotides. Let ∑_*H *_be the alphabet of symbols corresponding to higher-order elements that can be annotated over a genomic sequence, for instance TFs. We define a generic mapping function as a procedure to translate a sequence of nucleotides *S *= *s*_1_*s*_2 _... *s*_*k *_where each nucleotide *s*_*i*_∈ ∑_*DNA*_, into a sequence of tuples *M *= *m*_1_*m*_2 _... *m*_*n *_where each tuple mi=<mif,mip1,mip2,mis>
 MathType@MTEF@5@5@+=feaafiart1ev1aaatCvAUfKttLearuWrP9MDH5MBPbIqV92AaeXatLxBI9gBaebbnrfifHhDYfgasaacH8akY=wiFfYdH8Gipec8Eeeu0xXdbba9frFj0=OqFfea0dXdd9vqai=hGuQ8kuc9pgc9s8qqaq=dirpe0xb9q8qiLsFr0=vr0=vr0dc8meaabaqaciaacaGaaeqabaqabeGadaaakeaacqWGTbqBdaWgaaWcbaGaemyAaKgabeaakiabg2da9iabgYda8iabd2gaTnaaDaaaleaacqWGPbqAaeaacqWGMbGzaaGccqGGSaalcqWGTbqBdaqhaaWcbaGaemyAaKgabaGaemiCaaNaeGymaedaaOGaeiilaWIaemyBa02aa0baaSqaaiabdMgaPbqaaiabdchaWjabikdaYaaakiabcYcaSiabd2gaTnaaDaaaleaacqWGPbqAaeaacqWGZbWCaaGccqGH+aGpaaa@489E@ denotes the match of a motif for the higher-order element mif
 MathType@MTEF@5@5@+=feaafiart1ev1aaatCvAUfKttLearuWrP9MDH5MBPbIqV92AaeXatLxBI9gBaebbnrfifHhDYfgasaacH8akY=wiFfYdH8Gipec8Eeeu0xXdbba9frFj0=OqFfea0dXdd9vqai=hGuQ8kuc9pgc9s8qqaq=dirpe0xb9q8qiLsFr0=vr0=vr0dc8meaabaqaciaacaGaaeqabaqabeGadaaakeaacqWGTbqBdaqhaaWcbaGaemyAaKgabaGaemOzaygaaaaa@30EC@ ∈ ∑_*H *_occurring between the position mip1
 MathType@MTEF@5@5@+=feaafiart1ev1aaatCvAUfKttLearuWrP9MDH5MBPbIqV92AaeXatLxBI9gBaebbnrfifHhDYfgasaacH8akY=wiFfYdH8Gipec8Eeeu0xXdbba9frFj0=OqFfea0dXdd9vqai=hGuQ8kuc9pgc9s8qqaq=dirpe0xb9q8qiLsFr0=vr0=vr0dc8meaabaqaciaacaGaaeqabaqabeGadaaakeaacqWGTbqBdaqhaaWcbaGaemyAaKgabaGaemiCaaNaeGymaedaaaaa@31F0@ and the position mip2
 MathType@MTEF@5@5@+=feaafiart1ev1aaatCvAUfKttLearuWrP9MDH5MBPbIqV92AaeXatLxBI9gBaebbnrfifHhDYfgasaacH8akY=wiFfYdH8Gipec8Eeeu0xXdbba9frFj0=OqFfea0dXdd9vqai=hGuQ8kuc9pgc9s8qqaq=dirpe0xb9q8qiLsFr0=vr0=vr0dc8meaabaqaciaacaGaaeqabaqabeGadaaakeaacqWGTbqBdaqhaaWcbaGaemyAaKgabaGaemiCaaNaeGOmaidaaaaa@31F2@ over the sequence *S *with score mis
 MathType@MTEF@5@5@+=feaafiart1ev1aaatCvAUfKttLearuWrP9MDH5MBPbIqV92AaeXatLxBI9gBaebbnrfifHhDYfgasaacH8akY=wiFfYdH8Gipec8Eeeu0xXdbba9frFj0=OqFfea0dXdd9vqai=hGuQ8kuc9pgc9s8qqaq=dirpe0xb9q8qiLsFr0=vr0=vr0dc8meaabaqaciaacaGaaeqabaqabeGadaaakeaacqWGTbqBdaqhaaWcbaGaemyAaKgabaGaem4Camhaaaaa@3106@.

We introduced the concept of mapping for the promoter characterization problem in a previous work [9]. Let ∑_*TF *_be the alphabet of TFs denoting symbols. A mapping function is a procedure to translate a promoter region *S *= *s*_1_*s*_2 _... *s*_*k *_where each nucleotide *s*_*i *_∈ ∑_*DNA*_, into a sequence of TF-tuples *M *= *m*_1_*m*_2 _... *m*_*n *_where each TF-tuple mi=<mif,mip1,mip2,mis>
 MathType@MTEF@5@5@+=feaafiart1ev1aaatCvAUfKttLearuWrP9MDH5MBPbIqV92AaeXatLxBI9gBaebbnrfifHhDYfgasaacH8akY=wiFfYdH8Gipec8Eeeu0xXdbba9frFj0=OqFfea0dXdd9vqai=hGuQ8kuc9pgc9s8qqaq=dirpe0xb9q8qiLsFr0=vr0=vr0dc8meaabaqaciaacaGaaeqabaqabeGadaaakeaacqWGTbqBdaWgaaWcbaGaemyAaKgabeaakiabg2da9iabgYda8iabd2gaTnaaDaaaleaacqWGPbqAaeaacqWGMbGzaaGccqGGSaalcqWGTbqBdaqhaaWcbaGaemyAaKgabaGaemiCaaNaeGymaedaaOGaeiilaWIaemyBa02aa0baaSqaaiabdMgaPbqaaiabdchaWjabikdaYaaakiabcYcaSiabd2gaTnaaDaaaleaacqWGPbqAaeaacqWGZbWCaaGccqGH+aGpaaa@489E@ denotes the match of a binding site for the TF mif
 MathType@MTEF@5@5@+=feaafiart1ev1aaatCvAUfKttLearuWrP9MDH5MBPbIqV92AaeXatLxBI9gBaebbnrfifHhDYfgasaacH8akY=wiFfYdH8Gipec8Eeeu0xXdbba9frFj0=OqFfea0dXdd9vqai=hGuQ8kuc9pgc9s8qqaq=dirpe0xb9q8qiLsFr0=vr0=vr0dc8meaabaqaciaacaGaaeqabaqabeGadaaakeaacqWGTbqBdaqhaaWcbaGaemyAaKgabaGaemOzaygaaaaa@30EC@ ∈ ∑_*TF *_occurring between the position mip1
 MathType@MTEF@5@5@+=feaafiart1ev1aaatCvAUfKttLearuWrP9MDH5MBPbIqV92AaeXatLxBI9gBaebbnrfifHhDYfgasaacH8akY=wiFfYdH8Gipec8Eeeu0xXdbba9frFj0=OqFfea0dXdd9vqai=hGuQ8kuc9pgc9s8qqaq=dirpe0xb9q8qiLsFr0=vr0=vr0dc8meaabaqaciaacaGaaeqabaqabeGadaaakeaacqWGTbqBdaqhaaWcbaGaemyAaKgabaGaemiCaaNaeGymaedaaaaa@31F0@ and the position mip2
 MathType@MTEF@5@5@+=feaafiart1ev1aaatCvAUfKttLearuWrP9MDH5MBPbIqV92AaeXatLxBI9gBaebbnrfifHhDYfgasaacH8akY=wiFfYdH8Gipec8Eeeu0xXdbba9frFj0=OqFfea0dXdd9vqai=hGuQ8kuc9pgc9s8qqaq=dirpe0xb9q8qiLsFr0=vr0=vr0dc8meaabaqaciaacaGaaeqabaqabeGadaaakeaacqWGTbqBdaqhaaWcbaGaemyAaKgabaGaemiCaaNaeGOmaidaaaaa@31F2@ over the sequence *S *with score mis
 MathType@MTEF@5@5@+=feaafiart1ev1aaatCvAUfKttLearuWrP9MDH5MBPbIqV92AaeXatLxBI9gBaebbnrfifHhDYfgasaacH8akY=wiFfYdH8Gipec8Eeeu0xXdbba9frFj0=OqFfea0dXdd9vqai=hGuQ8kuc9pgc9s8qqaq=dirpe0xb9q8qiLsFr0=vr0=vr0dc8meaabaqaciaacaGaaeqabaqabeGadaaakeaacqWGTbqBdaqhaaWcbaGaemyAaKgabaGaem4Camhaaaaa@3106@.

**Figure 1:**
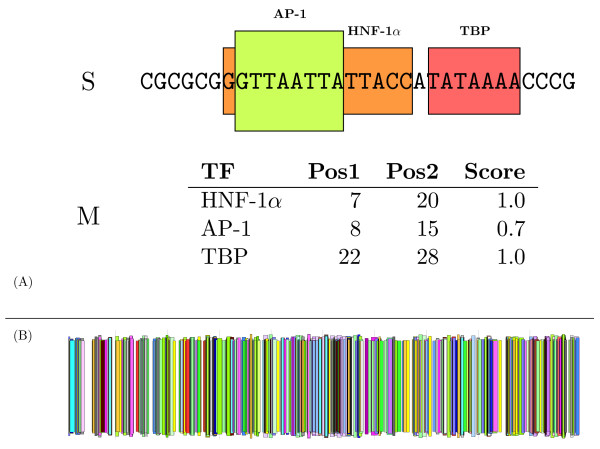
(A) The sequence of a promoter is searched for occurrences of known binding motifs for transcription factors (TFs), using a collection of position weight matrices. Matches are denoted as TF-tuples that contain the label of the TF, the positions of the match in the primary sequence, and the score provided by the corresponding matrix. Because binding sites tolerate sequence substitutions, several overlapping TF-tuples can be identified in a subset of positions. TF-tuples are graphically represented as boxes along the promoter sequence. Each TF is denoted with a different color. (B) Promoter TF-mapping of the *phospholipase A*1 gene (RefSeq: NM 015900, 500 nucleotides). TRANSFAC 6.3 was used as the mapping function [16]. In both cases, the TF occurrences are displayed as boxes along the promoter. In (A), the boxes are condensed in a single line. The TF-map graphical representation has been produced with the program gff2ps [27].

In the context of the detection of regulatory elements, different mapping functions can be used to obtain the translation from *S *to *M *such as a collection of position weight matrices (PWMs) representing TFBSs (JASPAR [14], PROMO [15] or TRANSFAC [16]), or a pattern discovery tool that identifies a set of unknown motifs conserved in several promoters (e.g. MEME [17]). In practice, for each match over a given threshold, we register a new TF-tuple in *M *defined by the label (mif
 MathType@MTEF@5@5@+=feaafiart1ev1aaatCvAUfKttLearuWrP9MDH5MBPbIqV92AaeXatLxBI9gBaebbnrfifHhDYfgasaacH8akY=wiFfYdH8Gipec8Eeeu0xXdbba9frFj0=OqFfea0dXdd9vqai=hGuQ8kuc9pgc9s8qqaq=dirpe0xb9q8qiLsFr0=vr0=vr0dc8meaabaqaciaacaGaaeqabaqabeGadaaakeaacqWGTbqBdaqhaaWcbaGaemyAaKgabaGaemOzaygaaaaa@30EC@) of the TF or the pattern, the positions (mip1
 MathType@MTEF@5@5@+=feaafiart1ev1aaatCvAUfKttLearuWrP9MDH5MBPbIqV92AaeXatLxBI9gBaebbnrfifHhDYfgasaacH8akY=wiFfYdH8Gipec8Eeeu0xXdbba9frFj0=OqFfea0dXdd9vqai=hGuQ8kuc9pgc9s8qqaq=dirpe0xb9q8qiLsFr0=vr0=vr0dc8meaabaqaciaacaGaaeqabaqabeGadaaakeaacqWGTbqBdaqhaaWcbaGaemyAaKgabaGaemiCaaNaeGymaedaaaaa@31F0@, mip2
 MathType@MTEF@5@5@+=feaafiart1ev1aaatCvAUfKttLearuWrP9MDH5MBPbIqV92AaeXatLxBI9gBaebbnrfifHhDYfgasaacH8akY=wiFfYdH8Gipec8Eeeu0xXdbba9frFj0=OqFfea0dXdd9vqai=hGuQ8kuc9pgc9s8qqaq=dirpe0xb9q8qiLsFr0=vr0=vr0dc8meaabaqaciaacaGaaeqabaqabeGadaaakeaacqWGTbqBdaqhaaWcbaGaemyAaKgabaGaemiCaaNaeGOmaidaaaaa@31F2@) and the score (mis
 MathType@MTEF@5@5@+=feaafiart1ev1aaatCvAUfKttLearuWrP9MDH5MBPbIqV92AaeXatLxBI9gBaebbnrfifHhDYfgasaacH8akY=wiFfYdH8Gipec8Eeeu0xXdbba9frFj0=OqFfea0dXdd9vqai=hGuQ8kuc9pgc9s8qqaq=dirpe0xb9q8qiLsFr0=vr0=vr0dc8meaabaqaciaacaGaaeqabaqabeGadaaakeaacqWGTbqBdaqhaaWcbaGaemyAaKgabaGaem4Camhaaaaa@3106@) of the match (see Figure 1(A), for an example). This translation preserves the order of *S *in *M*, that is if *i *<*j *in *M *then mip1
 MathType@MTEF@5@5@+=feaafiart1ev1aaatCvAUfKttLearuWrP9MDH5MBPbIqV92AaeXatLxBI9gBaebbnrfifHhDYfgasaacH8akY=wiFfYdH8Gipec8Eeeu0xXdbba9frFj0=OqFfea0dXdd9vqai=hGuQ8kuc9pgc9s8qqaq=dirpe0xb9q8qiLsFr0=vr0=vr0dc8meaabaqaciaacaGaaeqabaqabeGadaaakeaacqWGTbqBdaqhaaWcbaGaemyAaKgabaGaemiCaaNaeGymaedaaaaa@31F0@ <mjp1
 MathType@MTEF@5@5@+=feaafiart1ev1aaatCvAUfKttLearuWrP9MDH5MBPbIqV92AaeXatLxBI9gBaebbnrfifHhDYfgasaacH8akY=wiFfYdH8Gipec8Eeeu0xXdbba9frFj0=OqFfea0dXdd9vqai=hGuQ8kuc9pgc9s8qqaq=dirpe0xb9q8qiLsFr0=vr0=vr0dc8meaabaqaciaacaGaaeqabaqabeGadaaakeaacqWGTbqBdaqhaaWcbaGaemOAaOgabaGaemiCaaNaeGymaedaaaaa@31F2@. Matches to different TFs may possibly occur at the same position, being false positives in most cases (see a real example in Figure 1(B)). We refer to the resulting sequence of TF-tuples *M *as a Transcription Factor Map, or simply a TF-map.

In the implementation here, matches to PWMs are considered strandless, that is, they are annotated at a given location, irrespective of the orientation in which they occur. While biological evidence suggests that some TFBSs are functional only when present in a given strand, in other cases TF activity appears to be independent of the orientation of the binding site [18,19]. Since in general, we do not have information of the strand in which a binding site may be functional, we have not considered strand in our analysis.

### Multiple alignment of TF-maps

Let *M*_1_, *M*_2_, ..., *M*_*k *_be a set of TF-maps. Each map is denoted as *M*_*i *_= *m*_*i*, 1_*m*_*i*, 2 _... mi,|Mi|
 MathType@MTEF@5@5@+=feaafiart1ev1aaatCvAUfKttLearuWrP9MDH5MBPbIqV92AaeXatLxBI9gBaebbnrfifHhDYfgasaacH8akY=wiFfYdH8Gipec8Eeeu0xXdbba9frFj0=OqFfea0dXdd9vqai=hGuQ8kuc9pgc9s8qqaq=dirpe0xb9q8qiLsFr0=vr0=vr0dc8meaabaqaciaacaGaaeqabaqabeGadaaakeaacqWGTbqBdaWgaaWcbaGaemyAaKMaeiilaWIaeiiFaWNaemyta00aaSbaaWqaaiabdMgaPbqabaWccqGG8baFaeqaaaaa@362C@ where mi,jf
 MathType@MTEF@5@5@+=feaafiart1ev1aaatCvAUfKttLearuWrP9MDH5MBPbIqV92AaeXatLxBI9gBaebbnrfifHhDYfgasaacH8akY=wiFfYdH8Gipec8Eeeu0xXdbba9frFj0=OqFfea0dXdd9vqai=hGuQ8kuc9pgc9s8qqaq=dirpe0xb9q8qiLsFr0=vr0=vr0dc8meaabaqaciaacaGaaeqabaqabeGadaaakeaacqWGTbqBdaqhaaWcbaGaemyAaKMaeiilaWIaemOAaOgabaGaemOzaygaaaaa@3329@ ∈ ∑_*TF*_. Let M1∗,M2∗,...,Mk∗
 MathType@MTEF@5@5@+=feaafiart1ev1aaatCvAUfKttLearuWrP9MDH5MBPbIqV92AaeXatLxBI9gBaebbnrfifHhDYfgasaacH8akY=wiFfYdH8Gipec8Eeeu0xXdbba9frFj0=OqFfea0dXdd9vqai=hGuQ8kuc9pgc9s8qqaq=dirpe0xb9q8qiLsFr0=vr0=vr0dc8meaabaqaciaacaGaaeqabaqabeGadaaakeaacqWGnbqtdaqhaaWcbaGaeGymaedabaGaey4fIOcaaOGaeiilaWIaemyta00aa0baaSqaaiabikdaYaqaaiabgEHiQaaakiabcYcaSiabc6caUiabc6caUiabc6caUiabcYcaSiabd2eannaaDaaaleaacqWGRbWAaeaacqGHxiIkaaaaaa@3C0A@ be the extended set of TF-maps. Each extended map is denoted as Mi∗=mi,1∗,mi,2∗...mi,|Mi∗|∗
 MathType@MTEF@5@5@+=feaafiart1ev1aaatCvAUfKttLearuWrP9MDH5MBPbIqV92AaeXatLxBI9gBaebbnrfifHhDYfgasaacH8akY=wiFfYdH8Gipec8Eeeu0xXdbba9frFj0=OqFfea0dXdd9vqai=hGuQ8kuc9pgc9s8qqaq=dirpe0xb9q8qiLsFr0=vr0=vr0dc8meaabaqaciaacaGaaeqabaqabeGadaaakeaacqWGnbqtdaqhaaWcbaGaemyAaKgabaGaey4fIOcaaOGaeyypa0JaemyBa02aa0baaSqaaiabdMgaPjabcYcaSiabigdaXaqaaiabgEHiQaaakiabcYcaSiabd2gaTnaaDaaaleaacqWGPbqAcqGGSaalcqaIYaGmaeaacqGHxiIkaaGccqGGUaGlcqGGUaGlcqGGUaGlcqWGTbqBdaqhaaWcbaGaemyAaKMaeiilaWIaeiiFaWNaemyta00aa0baaWqaaiabdMgaPbqaaiabgEHiQaaaliabcYha8bqaaiabgEHiQaaaaaa@4BAC@ where mi,j∗f
 MathType@MTEF@5@5@+=feaafiart1ev1aaatCvAUfKttLearuWrP9MDH5MBPbIqV92AaeXatLxBI9gBaebbnrfifHhDYfgasaacH8akY=wiFfYdH8Gipec8Eeeu0xXdbba9frFj0=OqFfea0dXdd9vqai=hGuQ8kuc9pgc9s8qqaq=dirpe0xb9q8qiLsFr0=vr0=vr0dc8meaabaqaciaacaGaaeqabaqabeGadaaakeaacqWGTbqBdaqhaaWcbaGaemyAaKMaeiilaWIaemOAaOgabaGaey4fIOIaemOzaygaaaaa@3418@ ∈ *M*_*i*_∪ {-}. The symbol '-' indicates a gap, which can be considered as a particular TF-tuple < '-', · , ·, *γ *>. The value · denotes a null value, *γ *is the penalty for introducing a gap in a column of the alignment.

The multiple alignment of *k *maps *M*_1_, *M*_2_, ..., *M*_*k *_is defined as a rectangular array *T *where each column T(i)=(m1,i∗,m2,i∗,...,mk,i∗)
 MathType@MTEF@5@5@+=feaafiart1ev1aaatCvAUfKttLearuWrP9MDH5MBPbIqV92AaeXatLxBI9gBaebbnrfifHhDYfgasaacH8akY=wiFfYdH8Gipec8Eeeu0xXdbba9frFj0=OqFfea0dXdd9vqai=hGuQ8kuc9pgc9s8qqaq=dirpe0xb9q8qiLsFr0=vr0=vr0dc8meaabaqaciaacaGaaeqabaqabeGadaaakeaacqWGubavcqGGOaakcqWGPbqAcqGGPaqkcqGH9aqpcqGGOaakcqWGTbqBdaqhaaWcbaGaeGymaeJaeiilaWIaemyAaKgabaGaey4fIOcaaOGaeiilaWIaemyBa02aa0baaSqaaiabikdaYiabcYcaSiabdMgaPbqaaiabgEHiQaaakiabcYcaSiabc6caUiabc6caUiabc6caUiabcYcaSiabd2gaTnaaDaaaleaacqWGRbWAcqGGSaalcqWGPbqAaeaacqGHxiIkaaGccqGGPaqkaaa@4A7B@ is the multiple match among the TF-tuples in position *i *from the extended maps M1∗,M2∗,...,Mk∗
 MathType@MTEF@5@5@+=feaafiart1ev1aaatCvAUfKttLearuWrP9MDH5MBPbIqV92AaeXatLxBI9gBaebbnrfifHhDYfgasaacH8akY=wiFfYdH8Gipec8Eeeu0xXdbba9frFj0=OqFfea0dXdd9vqai=hGuQ8kuc9pgc9s8qqaq=dirpe0xb9q8qiLsFr0=vr0=vr0dc8meaabaqaciaacaGaaeqabaqabeGadaaakeaacqWGnbqtdaqhaaWcbaGaeGymaedabaGaey4fIOcaaOGaeiilaWIaemyta00aa0baaSqaaiabikdaYaqaaiabgEHiQaaakiabcYcaSiabc6caUiabc6caUiabc6caUiabcYcaSiabd2eannaaDaaaleaacqWGRbWAaeaacqGHxiIkaaaaaa@3C0A@:

T=(m1,1∗m1,2∗…m1,t∗m2,1∗m2,2∗…m2,t∗……mk,1∗mk,2∗…mk,t∗).
 MathType@MTEF@5@5@+=feaafiart1ev1aaatCvAUfKttLearuWrP9MDH5MBPbIqV92AaeXatLxBI9gBaebbnrfifHhDYfgasaacH8akY=wiFfYdH8Gipec8Eeeu0xXdbba9frFj0=OqFfea0dXdd9vqai=hGuQ8kuc9pgc9s8qqaq=dirpe0xb9q8qiLsFr0=vr0=vr0dc8meaabaqaciaacaGaaeqabaqabeGadaaakeaacqWGubavcqGH9aqpdaqadaqaauaabeqaeqaaaaaabaGaemyBa02aa0baaSqaaiabigdaXiabcYcaSiabigdaXaqaaiabgEHiQaaaaOqaaiabd2gaTnaaDaaaleaacqaIXaqmcqGGSaalcqaIYaGmaeaacqGHxiIkaaaakeaacqWIMaYsaeaacqWGTbqBdaqhaaWcbaGaeGymaeJaeiilaWIaemiDaqhabaGaey4fIOcaaaGcbaGaemyBa02aa0baaSqaaiabikdaYiabcYcaSiabigdaXaqaaiabgEHiQaaaaOqaaiabd2gaTnaaDaaaleaacqaIYaGmcqGGSaalcqaIYaGmaeaacqGHxiIkaaaakeaacqWIMaYsaeaacqWGTbqBdaqhaaWcbaGaeGOmaiJaeiilaWIaemiDaqhabaGaey4fIOcaaaGcbaGaeSOjGSeabaaabaaabaGaeSOjGSeabaGaemyBa02aa0baaSqaaiabdUgaRjabcYcaSiabigdaXaqaaiabgEHiQaaaaOqaaiabd2gaTnaaDaaaleaacqWGRbWAcqGGSaalcqaIYaGmaeaacqGHxiIkaaaakeaacqWIMaYsaeaacqWGTbqBdaqhaaWcbaGaem4AaSMaeiilaWIaemiDaqhabaGaey4fIOcaaaaaaOGaayjkaiaawMcaaiabc6caUaaa@6987@

Such a multiple TF-map alignment – or simply, a multiple map alignment (MMA), in contrast to a multiple sequence alignment (MSA) – satisfies the following conditions:

1. The extended maps have the same length.

2. If the gaps are removed from each Mi∗
 MathType@MTEF@5@5@+=feaafiart1ev1aaatCvAUfKttLearuWrP9MDH5MBPbIqV92AaeXatLxBI9gBaebbnrfifHhDYfgasaacH8akY=wiFfYdH8Gipec8Eeeu0xXdbba9frFj0=OqFfea0dXdd9vqai=hGuQ8kuc9pgc9s8qqaq=dirpe0xb9q8qiLsFr0=vr0=vr0dc8meaabaqaciaacaGaaeqabaqabeGadaaakeaacqWGnbqtdaqhaaWcbaGaemyAaKgabaGaey4fIOcaaaaa@3046@, the original *M*_*i *_is recovered.

3. At least one element in every column is different from a gap.

4. The elements that are aligned in a column correspond to the same TF.

Note that the first three conditions define the classical multiple alignment of sequences. The last one, however, introduces a new constrain that is related to the match state, according to the notion of pairwise TF-map alignment provided in [9].

### The score of a multiple alignment of TF-maps

Given the multiple alignment *T*, we compute the score of the MMA *s*(*T*) as:

α∑i=1t∑j=1kmj,i∗ss(T)=−λ(g)−μ∑∀i,i′f(m1,i∗p1−m1,i′∗p1,...,mk,i∗p1−mk,i′∗p1)
 MathType@MTEF@5@5@+=feaafiart1ev1aaatCvAUfKttLearuWrP9MDH5MBPbIqV92AaeXatLxBI9gBaebbnrfifHhDYfgasaacH8akY=wiFfYdH8Gipec8Eeeu0xXdbba9frFj0=OqFfea0dXdd9vqai=hGuQ8kuc9pgc9s8qqaq=dirpe0xb9q8qiLsFr0=vr0=vr0dc8meaabaqaciaacaGaaeqabaqabeGadaaakeaafaqaaeWaeaaaaeaaaeaaaeaaaeaaiiGacqWFXoqydaaeWaqaamaaqadabaGaemyBa02aa0baaSqaaiabdQgaQjabcYcaSiabdMgaPbqaaiabgEHiQiabdohaZbaaaeaacqWGQbGAcqGH9aqpcqaIXaqmaeaacqWGRbWAa0GaeyyeIuoaaSqaaiabdMgaPjabg2da9iabigdaXaqaaiabdsha0bqdcqGHris5aaGcbaGaem4CamNaeiikaGIaemivaqLaeiykaKcabaGaeyypa0dabaGaeyOeI0cabaGae83UdWMaeiikaGIaem4zaCMaeiykaKcabaaabaaabaGaeyOeI0cabaGae8hVd02aaabeaeaacqWGMbGzcqGGOaakcqWGTbqBdaqhaaWcbaGaeGymaeJaeiilaWIaemyAaKgabaGaey4fIOIaemiCaaNaeGymaedaaOGaeyOeI0IaemyBa02aa0baaSqaaiabigdaXiabcYcaSiqbdMgaPzaafaaabaGaey4fIOIaemiCaaNaeGymaedaaOGaeiilaWIaeiOla4IaeiOla4IaeiOla4IaeiilaWIaemyBa02aa0baaSqaaiabdUgaRjabcYcaSiabdMgaPbqaaiabgEHiQiabdchaWjabigdaXaaakiabgkHiTiabd2gaTnaaDaaaleaacqWGRbWAcqGGSaalcuWGPbqAgaqbaaqaaiabgEHiQiabdchaWjabigdaXaaakiabcMcaPaWcbaGaeyiaIiIaemyAaKMaeiilaWIafmyAaKMbauaaaeqaniabggHiLdaaaaaa@81D0@

where *α*, *λ*, *μ*, > 0, *g *is the number of columns with only one element different from a gap in the MMA (unaligned elements), and *f *is a function that measures the conservation of distance between the sites of every map in two consecutive columns (*i*, *i'*) with at least two aligned elements in the MMA. That is, the score of the alignment increases with the score of the aligned elements (*α*), and decreases with the number of gaps (*γ*), the number of unaligned elements (*λ*), and with the difference in the distance between adjacent aligned elements (*μ*). See [9] for further details about the TF-map alignment parameters.

### The algorithms

There are many possible alignments among multiple TF-maps. The optimal alignment is the one scoring the maximum (that is, showing maximum similarity) among all possible alignments. In a previous work [9], we implemented a dynamic programming algorithm to obtain such an alignment efficiently for the case of two TF-maps. The optimal multiple sequence alignment problem (and therefore the multiple alignment of maps as well) is, however, much more difficult, being formally a NP-complete problem [20].

Here, we propose to adapt the popular progressive alignment strategy to the TF-map alignment. The solutions obtained by this method are not guaranteed to be optimal. However, multiple progressive alignments usually capture the sequence features underlying the common functionality shared by the aligned sequences [5]. We have generalized the basic pairwise TF-map alignment algorithm developed in [9] in order to allow the comparisons between two single TF-maps, a TF-map and a MMA, and two MMAs.

### The alignment of two MMAs

Let *A*_*x *_= *m*_*x*, 1_*m*_*x*, 2 _... mx,|Ax|
 MathType@MTEF@5@5@+=feaafiart1ev1aaatCvAUfKttLearuWrP9MDH5MBPbIqV92AaeXatLxBI9gBaebbnrfifHhDYfgasaacH8akY=wiFfYdH8Gipec8Eeeu0xXdbba9frFj0=OqFfea0dXdd9vqai=hGuQ8kuc9pgc9s8qqaq=dirpe0xb9q8qiLsFr0=vr0=vr0dc8meaabaqaciaacaGaaeqabaqabeGadaaakeaacqWGTbqBdaWgaaWcbaGaemiEaGNaeiilaWIaeiiFaWNaemyqae0aaSbaaWqaaiabdIha4bqabaWccqGG8baFaeqaaaaa@3650@ and *A*_*y *_= *m*_*y*, 1_*m*_*y*, 2 _... my,|Ay|
 MathType@MTEF@5@5@+=feaafiart1ev1aaatCvAUfKttLearuWrP9MDH5MBPbIqV92AaeXatLxBI9gBaebbnrfifHhDYfgasaacH8akY=wiFfYdH8Gipec8Eeeu0xXdbba9frFj0=OqFfea0dXdd9vqai=hGuQ8kuc9pgc9s8qqaq=dirpe0xb9q8qiLsFr0=vr0=vr0dc8meaabaqaciaacaGaaeqabaqabeGadaaakeaacqWGTbqBdaWgaaWcbaGaemyEaKNaeiilaWIaeiiFaWNaemyqae0aaSbaaWqaaiabdMha5bqabaWccqGG8baFaeqaaaaa@3654@ be two MMAs that have been already computed. Let *S *be the scoring dynamic programming matrix where *S*(*i*, *j*) = *S*(*m*_*x*, *i*_, *m*_*y*, *j*_) denotes the score of the best TF-map alignment of the alignments *A*_*x *_= *m*_*x*, 1 _... *m*_*x*, *i *_and *A*_*y *_= *m*_*y*, 1 _... *m*_*y*, *j *_as defined previously (Equation 2). The *ComputePairwiseSimilarity *algorithm shown below is a generalization of that developed in [9] to align two TF-maps that computes the optimal pairwise TF-map alignment between *A*_*x *_and *A*_*y*_.

**Figure 2:**
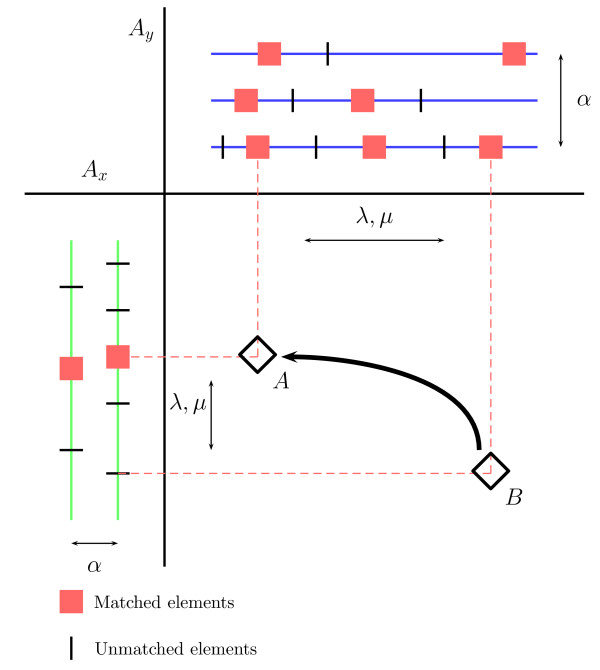
Graphical representation of the dynamic programming matrix produced in the alignment of two MMAs *A*_*x *_and *A*_*y*_. A new match between sites from both alignments must preserve the previous internal alignment (matches in red). For each new match (*A*), the best previous aligned match (*B*) must be selected to form the optimal alignment.

This algorithm basically searches the maps of both alignments to find matches between one site in the first alignment and one site in the second one. Once a new match is identified, the previous matches must be evaluated in order to construct the optimal alignment with this new one (see Figure 2). Because this class of scoring matrices are highly sparse [9], we register the coordinates in *S *of the matches computed previously. Thus, to compute the optimal score at the cell *S*(*i*, *j*), only the non-empty cells in *S *that are visible for the current match need to be accessed. In addition, we maintain the list sorted by optimal score, so that the cell scoring the maximum value is at the beginning of the list and, in most cases, only a few nodes will need to be accessed before a critical node is reached beyond which the optimal score can not be improved [9].

The number of computations *P*(*n*) in this algorithm is very similar to that obtained in the conventional pairwise TF-map alignment algorithm [9]. The exact complexity of this algorithm is difficult to be studied – depending mostly on the size of the input maps and the sparsity of the resulting matrix *S*. An expected time cost analysis reveals that the cost function can be explained in terms of (a) a first quadratic term derived from the necessary comparison between all of the TFBSs of both maps to detect the match cells and (b) a second quadratic term necessary to search for each match the best adjacent previous pair in the optimal TF-map alignment. In [9], we studied the positive contribution of using a list of non-empty cells in *S *that reduces the second component to an expected cost of *O*(*p*·*n*^2^), where *p *is the percentage of the similarity matrix that is occupied. In the case of pairwise TF-map promoter comparisons, this value was estimated to be below 0.05 (less than 5% of occupancy).

#### Implementation

In the pseudocode below, the alignments *A*_*x *_and *A*_*y *_are represented as two arrays of sites sorted by the position in their promoters, where each site corresponds to an input TFBS. The MMAs are internally encoded with pointers among the sites that form each match. Gaps here are not explicitly represented. Each site *m*_*x*, *i *_is a structure as described above with the functions *factor*, *pos1*, *pos2 *and *score *returning the values of the corresponding fields.

The variable *maxSim *stores the optimal score computed so far. The sites in the optimal TF-map alignment can be easily retrieved using a supplementary structure *path*(*i*, *j*) that points to the previous cell in the optimal path leading to cell *S*(*i*, *j*). In addition, the function *ComputeInitialSimilarity *calculates for each match *S*(*i*, *j*) the initial score of a hypothetical alignment that includes only the sites *m*_*x*, *i *_and *m*_*y*, *j*_. Once the match between two sites *m*_*x*, *i *_and *m*_*y*, *j *_has been identified, the best previous match between two other sites *m*_*x*, *i' *_and *m*_*y*, *j' *_is used to construct the new alignment (see the matches *A *and *B *in Figure 2). The list *L *is used to locate the non-empty positions in *S*. Each node of the list *L *is represented as structures *p *and *n *with the functions *abscissa *and *ordinate *returning the corresponding coordinates in *S *of each previous match.

The score of the new match between *m*_*x*, *i *_and *m*_*y*, *j *_is the sum of the scores of the columns in which both elements were aligned in their respective MMAs. Unaligned sites are scored with the gap penalty *γ *. The function *ComputeLambda *counts the number of sites in each group that are not included in the alignment, taking into account the size of each individual MMA.

In practice, we do not allow overlap in the primary sequence between adjacent sites in the alignment. This is not a practical limitation of the algorithm, but a requirement introduced according to our observations in available annotations of regulatory elements. The function *ComputeOverlap *calculates the average distances *D*1 and *D*2 between any pair of consecutive matches in the maps of each alignment, verifying the absence of physical overlap in their promoters. The function |*D*1 - *D*2| scores the conservation of distance between the sites of every map in two consecutive columns on each MMA (function *f*, see Equation 2).

#### ComputePairwiseSimilarity

**Require: ***A*_*x*_, *A*_*y*_: TF-map alignments, *L*: list of <abscissa, ordinate>, *L *= ∅

   {Calculating the element *i*, *j *in *S*}

      **for ***i *= 0 to |*A*_*x*_*| - *1 **do**

         **for ***j *= 0 to |*A*_*y*_*| *- 1 **do**

            **if **factor(*m*_*x*, *i*_) = factor(*m*_*y*, *j*_) **then **

5:             *S*(*i*, *j*)← ComputeInitialSimilarity(*m*_*x*, *i*_, *m*_*y*, *j*_);

               *x *← *α *(score(*m*_*i*_) + score(*m*_*j*_));

               {Searching the best previous match in *L*}

               *p *← first (*L*);

               *i' *← abscissa(*p*);

10:            *j' *← ordinate(*p*);

               **while **end(*L*) = FALSE and *S*(*i'*, *j'*)*+ x *>*S*(*i*, *j*) **do**

                  {Compute the *μ *value and check overlap}

                  (*D*1, *D*2, overlap) ← ComputeOverlap(*i*, *i'*, *j*, *j'*, *A*_*x*_, *A*_*y*_);

                  **if **overlap = FALSE **then**

15:               *y *← *λ *(ComputeLambda(*i*, *i*', *j*, *j'*));

                  *z *← *μ *(|*D*1- *D*2|);

                  maxSim ← *S*(*i'*, *j'*) + *x - y - z;*

                  **if **maxSim > *S*(*i*, *j*)**then**

                     *S*(*i*, *j*) ← maxSim;

20:            *p *← next(*L*);

               *i' *← abscissa(*p*);

               *j' *← ordinate(*p*);

            *n *← CreateNewNode(*i*, *j*);

            InsertNode(*n*, *L*);

### Progressive MMA algorithm

Let (*A*_1 _... *A*_*k*_) be the initial list of *k *TF-map alignments, where each alignment *A*_*i *_contains a single TF-map. Let *S *be the similarity matrix where *S*(*A*_*i*_, *A*_*j*_) denotes the similarity between two TF-map alignments *A*_*i *_and *A*_*j*_.

The progressive MMA algorithm shown below builds up a multiple TF-map alignment in a stepwise manner. In a first step, all pairwise TF-map alignments are performed. The initial multiple alignment is created with the two most similar ones. These maps are substituted for the alignment of both. The similarity between this new alignment and the rest of the TF-maps is then estimated, updating the *S *matrix (see Implementation).

In a second step, an iterative procedure selects at each round the pair of alignments that are more similar from the pool of available ones. These two alignments are then merged into a new MMA, estimating the similarity to the remaining ones. At the end of the process, there is only one alignment that contains the multiple alignment of the input maps.

The cost of the progressive MMA can be expressed in terms of the number of pairwise TF-map alignments that must be computed. Let *k *be the number of maps to be aligned and *n *be the length of each map. The initial round performs *O*(*k*^2^) pairwise alignments. Next, the progressive rounds perform *O*(*k*) alignments involving two previous alignments. Let *P*(*n*) be the cost of each pairwise operation (see previous section), then the cost of the progressive map alignment algorithm is *O*(*k*^2 ^*P*(*n*)).

#### Implementation

In the progressive MMA algorithm shown below, the variable *maxSim *saves the maximum score so far computed at each round. The identifiers of the alignments that produce such a score can easily be retrieved using a supplementary pair of variables *iSim*, *jSim*.

The function *ComputePairwiseSimilarity *is a generalization of the TF-map alignment algorithm presented in [9], as explained in the previous section. The optimal pairwise alignments between the input TF-maps in the initial round are saved, as they could be required during the iterative procedure.

Once a new TF-map alignment is created from the two most similar ones, their binding sites must be merged (function *MergeAlignments*). The order of the TFBSs in the new alignment must take into account the position of the binding sites in their primary promoter sequences. In the approach here, we do not create a profile of each MMA. Instead, all of the TFBSs of each alignment are always available for subsequent TF-map alignments.

The alignments between this new TF-map alignment and the others are not explicitly computed. The similarity of them is instead estimated with the WPGMA method (Weighted Pair Group Method with Arithmetic Mean [21]), in which the similarity of the previous alignments between *A*_*iSim *_and *A*_*jSim *_to the other alignments is weighted according to the number of maps of each one. If an estimated alignment between two MMAs is identified as the most similar one during the progressive step, then it must be explicitly computed before merging both TF-map alignments.

#### Progressive MMA algorithm

**Require: ***A*: list of TF-map alignments (*A*_1 _... *A*_*k*_)

   {Initial Step: pairwise alignment all Vs all}

   maxSim ← -∞

   **for ***i *= 1 to *k ***do**

5:  **for ***j *= *i *+ 1 to *k ***do**

         *S*(*A*_*i*_*, A*_*j*_) ← ComputePairwiseSimilarity(*A*_*i*_, *A*_*j*_);

         {Select the pair with maximum similarity}

         maxSim ← max(maxSim, *S*(*A*_*i*_, *A*_*j*_));

   {Create a new MMA: estimate the similarity to others}

10:*A*_*iSim*-*jSim *_← Merge Alignments(*A*_*iSim*_*, A*_*jSim*_);

   {Progressive Step: select the two most similar alignments}

   **while **|*A*| > 1 **do**

      maxSim ← -∞

15:   **for ***i *= 1 to |*A*| **do**

         **for ***j *= *i *+ 1 to |*A*| **do**

            {Select the pair with maximum similarity}

            maxSim ← max(maxSim, *S*(*A*_*i*_, *A*_*j*_));

      {Create a new MMA: estimate the similarity to others}

20:   *A*_*iSim*-*jSim *_← Merge Alignments (*A*_*iSim*_, *A*_*jSim*_);

### Non-collinear TF-map alignments

The existence of regulatory elements that are conserved in different order among related regulatory regions has been documented in a few cases, specially in enhancers [22]. The identification of these regulatory rearrangements is very difficult at the sequence level. We have here introduced some subtle changes in the pairwise TF-map alignment algorithm shown before to deal with non-collinear alignments. The aligned TFBSs in such MMAs are therefore not necessarily located in the same relative order in every map.

#### Definition

**Figure 3:**
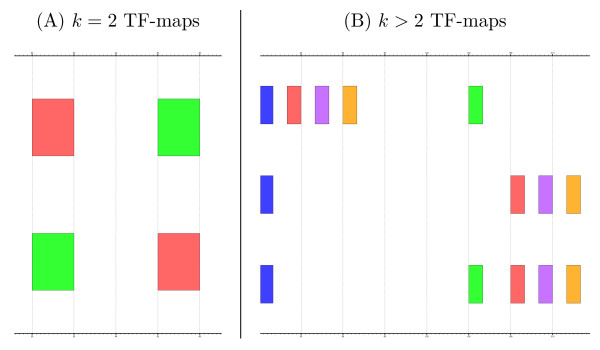
Graphical representation of two examples of non-collinear TF-map alignments. In (A), the alignment involves only two TF-maps. In (B), the resulting multiple alignment of three maps contains a non-collinear block of three TFBSs.

Let T be an alignment between two TF-maps *M*_1 _and *M*_2 _formally defined as a correspondence *T *= {(*m*_1, 1_, *m*_2, 1_), ..., (*m*_1, *t*_, *m*_2, *t*_)} [9]. Let (*m*_1, *i*_, *m*_2, *j*_) and (*m*_1, *k*_, *m*_2, *l*_) be two matches in *T*, not necessarily contiguous, with *i *<*k*. Then, we define the collinearity or non-collinearity of *T *in terms of the partial order between *j *and *l*, for all the match pairs of *T *as:

1. If *j *<*l *then *T *is a collinear alignment

2. If *j *> *l *then *T *is a non-collinear alignment (see example shown in Figure 3(A)).

The generalization of this definition for *k *> 2 TF-maps is trivial (see the example of a non-collinear alignment for *k *= 3 TF-maps in Figure 3(B)).

#### Algorithm

**Figure 4:**
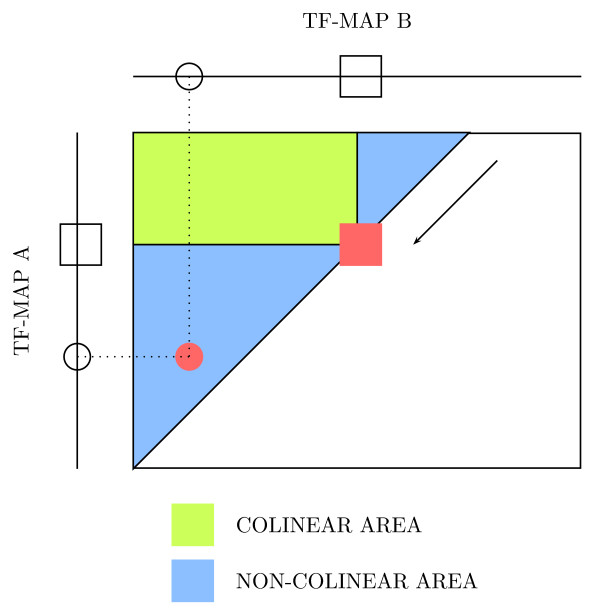
Different forms to fill a dynamic programming matrix in during the alignment. For a current match between two alignments (denoted as a red square), the available area to search the best previous match when the matrix is processed row by row is depicted in green. For the same element, the additional area to search the best previous match when the matrix is processed diagonal by diagonal is depicted in blue. With the diagonal filling, a previous match (denoted as a red circle) that forms a non-collinear alignment can be detected.

The non-collinear matches shown in Figure 3 can not be detected in the basic pairwise TF-map alignment algorithm [9]. Let *A *and *B *be two TF-maps in which two matches could form a non collinear alignment (represented as a circle and a square in Figure 4). The normal implementation fills the matrix in row by row, from top to bottom (or column by column, from left to right).

According to this, when the first match is being processed (red square), the second one (red circle) is not yet available (the red circle is not in the green area). Conversely, when the second match is processed, the first one is not accessible as the basic algorithm only allows the search for best previous aligned elements in the list of computed values that are in the area delimited by the current match (area denoted by dotted lines).

To overcome such a limitation, we propose to compute the optimal values of the matrix *S *following a different order, to allow the second element (red circle) to be visible when the first one is being processed (red square). A diagonal filling of the matrix calculates first the match between circles (see Figure 4).

Thus, this element will be available to compute the best alignment for the match between squares that is processed later. While this strategy still produces the same alignments obtained with the ordinary implementation, non-collinear alignments produced by new combinations of matches can also be constructed.

#### Adjusting the non-collinearity

We have designed a simple mechanism to adjust the frequency of non-collinear aligned sites in the output. An additional parameter *c *has been introduced in the basic MMA algorithm to weight those alignments involving non-collinearity.

**Figure 5:**
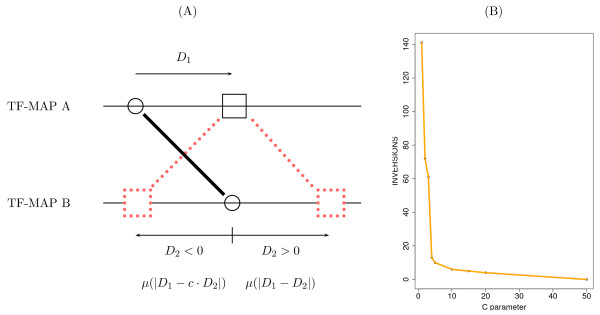
(A) A graphical explanation of the parameter *c *that controls the proportion of non-collinear blocks in a resulting alignment; (B) Number of inversions in the TF-map alignment of the human and mouse promoters (500 nucleotides) of the *MMP13 *gene (RefSeq: NM 002427 and NM 008607), using multiple values of *c*.

Let *A *and *B *be two TF-maps in which a previous match has been identified (represented as two circles in Figure 5(A)). Then, a second match between one element in *A *and another one in *B *is being processed (represented as two squares in Figure 5(A)). The dotted lines indicate that such a site in *B *can be located either on the left or on the right of the circle site in the same map. In the first case, a non-collinear alignment is produced; in the second case, a normal collinear alignment is constructed. The case in which the non-collinear match occurs in *A *can be similarly defined.

The algorithm to align two MMAs must be slightly modified to accomodate the non-collinearity parameter *c*. The variable *z *in the *ComputePairwiseSimilarity *is defined now as:

z={if(D2<0)→μ|D1−c⋅D2|,c≥1if(D2≥0)→μ|D1−D2|.
 MathType@MTEF@5@5@+=feaafiart1ev1aaatCvAUfKttLearuWrP9MDH5MBPbIqV92AaeXatLxBI9gBaebbnrfifHhDYfgasaacH8akY=wiFfYdH8Gipec8Eeeu0xXdbba9frFj0=OqFfea0dXdd9vqai=hGuQ8kuc9pgc9s8qqaq=dirpe0xb9q8qiLsFr0=vr0=vr0dc8meaabaqaciaacaGaaeqabaqabeGadaaakeaacqWG6bGEcqGH9aqpdaGabeqaauaabaqacmaaaeaacqqGPbqAcqqGMbGzaeaacqGGOaakcqWGebardaWgaaWcbaGaeGOmaidabeaakiabgYda8iabicdaWiabcMcaPaqaaiabgkziUIGaciab=X7aTjabcYha8jabdseaenaaBaaaleaacqaIXaqmaeqaaOGaeyOeI0Iaem4yamMaeyyXICTaemiraq0aaSbaaSqaaiabikdaYaqabaGccqGG8baFcqGGSaalcqWGJbWycqGHLjYScqaIXaqmaeaacqqGPbqAcqqGMbGzaeaacqGGOaakcqWGebardaWgaaWcbaGaeGOmaidabeaakiabgwMiZkabicdaWiabcMcaPaqaaiabgkziUkab=X7aTjabcYha8jabdseaenaaBaaaleaacqaIXaqmaeqaaOGaeyOeI0Iaemiraq0aaSbaaSqaaiabikdaYaqabaGccqGGPaqkaaGaeiOla4cacaGL7baaaaa@62EE@

The optimal positional conservation between both matches occurs when *D*_1 _= *D*_2_. However, the parameter c is used in the *μ*, penalty to punish only those matches that do not respect the collinearity of the current alignment (the square site is on the left of the circle site in *B*, see Figure 5(A)).

Informally, if *c *= 1 then both collinear and non-colinear matches are indistinctly combined into the resulting MMA. High values of *c*, however, produce a higher amount of collinear matches into the results. In order to establish formally the behaviour of this parameter, we have counted the number of non-collinear matches in the TF-map alignment of the human and mouse promoters (500 nucleotides) of the *MMP13 *gene (RefSeq: NM_002427, NM_008607). In Figure 5(B), there is a clear correspondence between the amount of inversions in the MMA and the value of *c*. No inversions are produced for large values of *c*. Identification of non-collinear configurations of TFBSs in regulatory regions is poorly known, and only a few cases are documented [22]. We recommend, therefore, to use this option very carefully. In addition, we also suggest the use of a small set of matrices to perform the mapping, which can reduce the number of artifacts in the resulting non-collinear MMA (see Promoter characterization section, *even-skipped *stripe 2 enhancer).

### Datasets and software availability

The datasets used in this paper are available at [23]. An implementation of this algorithm has been written in C and is publicly available at [24]. A web server that performs the mapping and the alignment of multiple promoter regions with such an algorithm is accessible at [25].

**Figure 6:**
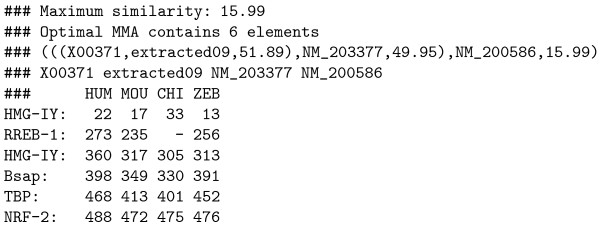
Results of the TF-map alignment of the human promoter of the *Myoglobin *gene and the orthologs in mouse, chicken and zebrafish. Here, the 500 nucleotides upstream of the annotated TSS have been considered (with position 1 corresponding to -500). Each row contains the aligned occurrences of the same TF at the TF-maps of some species (only the starting position of the binding site is shown). Gaps are represented with the symbol "-". A graphical representation of the TF-map alignment is shown in Figure 8.

The input of the program consists of a file that contains the TF-maps to be aligned. The file must be in General Feature Format [26]. Options allow to control the values of *α*, *λ*, *μ*, *γ *and *c *as well as to display the results in plain format or GFF format. The output includes the score and the length of the optimal MMA, and the matches in the input TF-maps (see Figure 6 for an example). A graphical representation of the MMA is also displayed using the program gff2ps [27].

## Results

The optimal MMA of a set of TF-maps is obviously dependant on the values of the *α*, *λ*, *μ*, *γ *and *c *parameters. In addition, the optimal parameter configuration is likely to depend on the particular problem to be addressed (orthologous genes or co-regulated genes in microarray experiments), and the particular protocol to map the TFBSs on the sequences.

Results in a previous work [9] indicated that TF-maps alignments are able to characterize promoter regions of co-regulated genes even in absence of sequence similarity. Thus, TF-map alignments were shown to detect high-order regulatory signals conserved in a collection of related promoters that were undetectable with current sequence alignment methods. It is important to mention that two or more different TFBSs can be aligned if and only if they correspond to the same TF, even though they may not show sequence similarity.

Here we have conducted a similar systematic training over an extended set of orthologous promoters to obtain the optimal parameter configuration. In order to verify the ability of MMA to identify regulatory elements that are rarely detected in conventional comparisons, we have compared the results to those obtained by several multiple sequence alignment methods. We have focused on two specific examples to show the abilities of MMA in the characterization of multiple collinear related promoters. Finally, we have characterized non-collinear arrangements of TFBSs on an early developmental enhancer conserved in several species of *Drosophila*.

### Multiple TF-map training

For the pairwise TF-map alignment, we estimated the optimal parameters in a set of experimentally characterized human and rodent gene promoters [9,13]. Here we have extended such a dataset by searching the corresponding orthologs in chicken and zebrafish as well. Using the RefSeq [28] gene set as mapped into the UCSC genome browser, we have correctly identified the ortholog in both species, if available. We refer to the resulting set of human-mouse-chicken-zebrafish homologous genes as the HRCZ SET. This dataset contains 18 human-rodent-chicken-zebrafish orthologs, 7 human-rodent-chicken orthologs, 4 human-rodent-zebrafish orthologs, and 7 human-rodent orthologs.

The lack of available collections of experimentally verified TFBSs is an important limitation for the evaluation and the training of phylogenetic footprinting systems. Despite several databases of annotations and promoter sequences have recently appeared [13,29], there is not enough regulatory information conserved among species other than human and mouse to train the MMA.

Thus, we can not repeat the training procedure used in [9] to evaluate the ability of MMA to detect conserved regulatory elements at larger evolutionary distances – at which the degree of conservation may be negligible. However, we can use another method also presented in [9] to show that MMAs are much more informative than primary multiple sequence alignments.

We first have mapped the TFBSs occurrences in the promoter sequences using the collection of 50 most informatives matrices in JASPAR 1.0 [14]. In a previous work [9], we observed a substantial gain of specificity in the detection of real TFBSs (without a significant loss of sensitivity) when using such a subset of matrices instead of the entire JASPAR collection. The original frequency coefficients of the matrices were converted before into log-likelihood ratios, to which we referred as JASPAR_*TOP*50 _in [9], using the random equiprobability distribution as a background model. A prediction obtained with a given PWM from JASPAR_*TOP*50 _was accepted if it had a score above 50% of the maximum possible score for such matrix [23].

**Figure 7:**
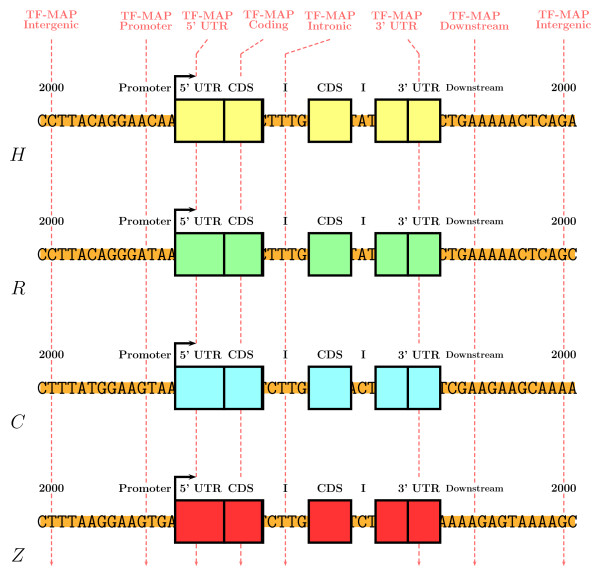
Graphical representation of the MMA training. For multiple combinations of *α*, *λ*, *μ *and *γ*, we have compared the multiple TF-map alignments obtained in the proximal promoter regions from the HRCZ SET (200 nucleotides), with the alignments obtained in fragments of 200 nucleotides from intergenic (2000 nucleotides upstream of the TSS and 2000 nucleotides downstream of the transcript), 5'UTR (downstream of the TSS), 3'UTR (upstream of the end of the transcript), coding (downstream of the translation start site and considering only coding DNA), intronic (downstream of the first intron junction), and downstream (downstream of the transcription termination site) sequences.

Then, we have compared the MMAs obtained in the 200 nucleotides of the promoter region of the 36 gene pairs from the HRCZ SET, with the MMAs obtained using the same mapping function in fragments of 200 nucleotides from intergenic (2000 nucleotides upstream of the TSS, 2000 nucleotides downstream the transcript), 5'UTR (downstream of the TSS), 3'UTR (upstream the end of the transcript), coding (downstream of the translation start site and considering only coding DNA), intronic (downstream of the first intron junction), and downstream (downstream of the transcription termination site) sequences (see Figure 7 for a graphical representation of the test).

We have computed the average score of the MMA on each one of the genomic regions, identifying the alignment that produces the highest score for each orthologous set. We have repeated this test using different combinations of parameters. Systematically, the parameters *α*, *λ*, and *μ *were allowed to independently take values between 0.0 and 1.0, in incremental steps of 0.1. At the same time, the parameter *γ *(gap penalty) was tested between 0 and -10 with a step of -1. The optimal parameter configuration is considered to be that set of parameter values that better discriminate between promoters and the rest of genomic regions.

We have also performed the multiple sequence alignments of the same regions using the following programs (see [30] for a comprehensive review on DNA sequence alignment): CLUSTALW [5], MLAGAN [31] and FOOTPRINTER [32]. CLUSTALW and MLAGAN perform global multiple sequence alignment.

FOOTPRINTER performs local multiple sequence alignment. The number of significant motifs (parsimony score 2) identified by FOOTPRINTER on each gene region was used to rank the alignments of this program.

Results appear in Table 1. As expected, nucleotide sequence alignments score the highest in the coding regions (in 27 out of 36 cases for CLUSTALW, 26 and 23 cases for MLAGAN and FOOTPRINTER respectively), followed by the alignments in the 5'UTRs (4 out of 36 for CLUSTALW, 4 and 3 for MLAGAN and FOOTPRINTER) and in the promoters (4 out of 36 for CLUSTALW, 4 and 5 for MLAGAN and FOOTPRINTER). Only in one case, the 3'UTR was the most conserved region among orthologs (in three cases for FOOTPRINTER). The scores of the sequence alignments indicate that promoter regions are less conserved than coding regions.

**Table 1:** Multiple alignment results in orthologous sequences

HRCZ SET	MMA		CLUSTALW		MLAGAN		FOOTPRINTER	
	TOP1	Avg.Score	TOP1	Avg. score	TOP1	Avg. score	TOP1	Avg. Motifs

Coding	6	17.15	27	3706.72	26	3739.58	23	20.41
5'UTR	2	10.48	4	2671.78	4	2742.92	3	11.66
Promoter	18	25.41	4	2005.67	4	2114.00	5	8.66
3'UTR	7	15.85	1	1994.22	1	2046.31	3	9.63
Intronic	2	8.34	0	1267.89	0	1268.42	1	3.00
Downstream	0	6.85	0	1174.28	1	1245.47	1	2.02
5'Intergenic	0	5.42	0	1052.92	0	1092.28	0	0.75
3'Intergenic	1	4.14	0	974.69	0	1027.58	0	0.44

TF-map and sequence alignments (CLUSTALW [5], MLAGAN [31], FOOTPRINTER [32]) of different genomic regions between the human, mouse, chicken and zebrafish orthologous promoters in the HRCZ SET. TOP1 is the number of genes in which the highest scoring alignment is found in a given genomic region. The MMA results were obtained with the optimal configuration *α *= 1; *λ *= 0.3; *μ *= 0.1; *γ *= 2.

Despite this, the optimal collinear MMA configuration [*α *= 1, *λ *= 0.3, *μ *= 0.1, *γ *= -2] scores the highest in the promoter regions (in 18 out of 36, see Table 1). In addition, the average score of map alignments is notably higher than that of the coding regions (25.41 against 17.15). Only in 6 out of 36 cases the TF-map alignments score the highest in coding regions. Interestingly, while 3'UTR sequences in the human-mouse-chicken-zebrafish orthologs are much less conserved than coding regions or 5'UTRs, MMAs score the highest in them in 7 cases. This is consistent with recent investigations about the existence of regulatory motifs in the 3'UTR regions of the genes [33]. A similar result is obtained in the case of introns: intronic sequences are much less conserved than coding and UTR sequences. However, MMAs score the highest in intronic regions in 2 cases. This fact is noticeable as first introns are also known to often contain regulatory motifs [34,35].

We have also performed a complementary test to measure the specificity of the TF-map alignments. As a negative control, we have shuffled the orthologous associations in the HRCZ SET to construct a pool of 36 unrelated human-mouse-chicken-zebrafish gene entries. Then, the corresponding multiple TF-map alignments of these non-orthologous paired promoters were obtained using the parameters previously optimized. Results appear in Table 2. The TF-map alignments of unrelated promoters were significantly worse with an average score more than 50% smaller than TF-map alignments that involved "bona fide" orthologous promoters. For instance, the average score of the TF-map alignments among orthologous promoters when using the JASPAR_*TOP*50 _collection was 25.41 (see Table 2). In contrast, the score of the TF-map alignments between non-related promoters was 9.91. The sites in the alignments involving non-orthologous gene promoters may correspond to general regulatory elements present in most core promoters of our dataset.

**Table 2:** Specificity of the TF-map alignments

MMA ON HRCZ SET	Avg	Avg*	Max	Max*
Coding	17.15	4.66	62.60	31.37
5'UTR	10.48	3.60	35.74	26.16
Promoter	25.41	9.91	71.22	46.18
3'UTR	15.85	7.23	76.31	50.51
Intronic	8.34	3.53	48.60	22.39
Downstream	6.85	4.88	24.91	24.10
5'Intergenic	5.42	3.65	21.36	24.12
3'Intergenic	4.14	4.53	35.70	25.40

Comparison between average and maximum scores of TF-map alignments of several regions from orthologous genes from the HRCZ SET and average and maximum scores of TF-map alignments of several regions from randomly shuffled genes from the HRCZ SET (denoted as *, see main text for details).

To validate this hypothesis, we have analyzed the composition of the TF-map alignments of non-orthologous gene promoters. We have detected an enrichment in TATA and CAAT boxes (20% and 10% of the aligned sites, respectively), which are well known to be part of core promoters. Such a bias is not observed in the composition of the alignments of the coding sequences of unrelated genes. These alignments are therefore partially capturing common regulatory elements present in unrelated gene promoters of our dataset. In addition, we also found an overrepresentation (25% of the aligned sites) of TFs mainly expressed in the liver (HNF3, COUP-TF, HNF1). Such a trend is not detected in the composition of the alignments of the coding sequences of non-related genes. This enrichment correlates well with the composition of the HRCZ SET, which contains experimental regulatory annotations from liver-specific genes (e.g. [36] and [37]; see [9] for further details).

We have performed an additional test to assess the significance of the scores of the MMAs. The previous tests have involved alignments of orthologous gene regions of the same type (e.g. four promoters or four coding segments). We have compared now the score of the MMAs among orthologous promoters of the same gene in the HRCZ SET with the scores of the alignments of the same maps in which one TF-map was randomly substituted by the TF-map of another segment of the same gene (denoted in Table 3 as *PPPS*, where *P *is a promoter map and *S *is any gene region: Coding, Promoter, Intronic, 5'UTR, 3'UTR, intergenic, downstream).

**Table 3:** Significance of the TF-map alignment scores

	S							
	Promoter	Coding	5'UTR	3'UTR	Intronic	Downstream	5'Intergenic	3'Intergenic

*PPPS*	25.41	10.06	7.43	5.36	10.54	10.84	10.84	10.77
*PPSS*	25.41	9.79	5.03	5.24	7.50	6.31	4.98	5.61
*P*_*H*_*S*_*R*_	42.00	5.80	5.04	2.49	4.57	5.79	4.64	3.63

Average score of multiple TF-map alignments of groups constituted of orthologous promoters from the HRCZ SET (denoted as *P*), introducing one or two non-corresponding orthologous sequences (denoted as *S*). Last row shows the average score of pairwise TF-map alignments that involve each human promoter (*P*_*H*_) and the corresponding rodent region of the same gene (*S*_*R*_) in the HRCZ SET.

Results appear in Table 3. The average score of MMAs exclusively constituted by promoter maps was 25.41 (*PPP + *Promoter in Table 3). Indeed, the average score of the MMAs involving only promoter maps was more than 60% higher than alignments in which one of them was substituted by another gene region map (e.g. 10.06 for *PPP + *Coding in Table 3). The average score of such alignments dropped even more when a second substitution was permitted (see Table 3).

Finally, we analyzed the scores of pairwise TF-map alignments between each human promoter in the HRCZ SET (*P*_*H*_) and the corresponding orthologous gene regions (*S*_*R*_) in mouse. The average score of the TF-map alignments involving the two promoters was substantially higher (42.00) than any other incorrect combination (e.g. 5.80 for human promoter-mouse coding region alignments). These results show that orthologous promoter-promoter TF-map alignments are more significant than alignments of any other combination of gene region maps.

### Promoter characterization

We have selected three particular examples that show the ability of MMAs to characterize promoter regions in the absence of sequence conservation. In all cases, we have compared the multiple TF-map alignment to the corresponding multiple sequence alignments, as in the section above, to measure their accuracy to detect the TFBSs experimentally annotated on these promoters.

#### Actin *α*-cardiac gene

Actins are highly conserved proteins that are involved in various types of cell motility. The alpha actins are found in muscle tissues and are a major constituent of the contractile apparatus. The *Actin α-cardiac *gene has been identified in many kinds of cells including muscle, where it is a major constituent of the thin filament, and platelets [38].

The promoter of the human and mouse *Actin α-cardiac *genes (ACTC, GenBank: M13483, M26773) has been extensively characterized by experimental means [39]. In the ABS database [13], the entry *A*0028 includes the known orthologous binding sites in the respective human and mouse promoters (500 nucleotides, the position +501 is the TSS). The human ACTC promoter is constituted of three SRF sites (+301, +352, +392), a SP1 site (+418), and a TATA box (+469).

Using the RefSeq gene annotations [28], we have also identified the corresponding orthologous promoters in chicken and zebrafish (RefSeq: NM_001031229, NM_214784).

We have then aligned the four promoters and compared the resulting MMA with the functional annotations in the ABS database. In general, the multiple TF-map alignment of the four orthologous promoters of ACTC contains many of the known functional sites in human and mouse, detecting as well the corresponding orthologs in the other species.

**Figure 8:**
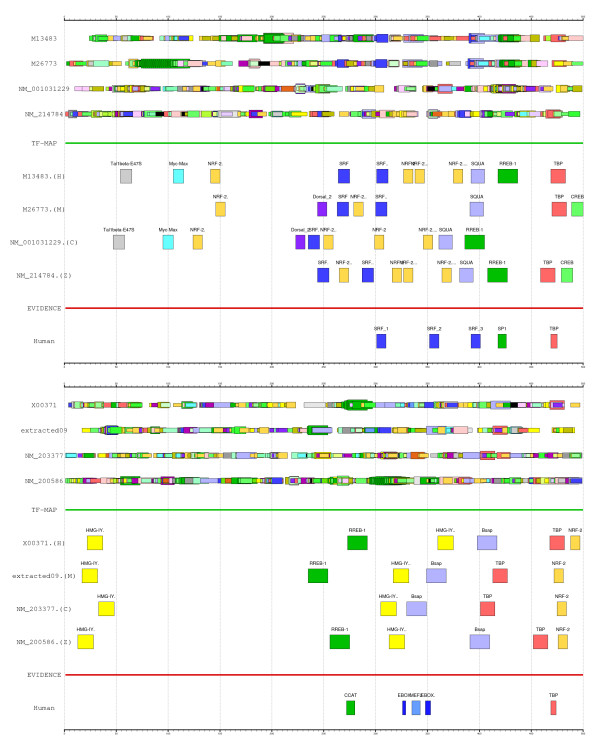
On top, the JASPAR predictions on the human-mouse-chicken-zebrafish promoters of the *Actin α-cardiac *gene (ACTC, GENBANK: M13483 and M26773, RefSeq: NM 001031229 and NM 214784), the resulting MMA and the experimental evidence. At bottom, the JASPAR predictions on the human-mouse-chicken-zebrafish promoters of the *Myoglobin *gene (MB, GENBANK: X00371, RefSeq: NM 013593, NM 203377 and NM 200586), the resulting MMA and the experimental evidence. In both cases, the TF-map alignment represents a considerable noise reduction which has biological relevance, as most experimentally annotated TFBSs in these promoters are successfully covered by the MMAs (see main text). Both graphical representations have been produced with the program gff2ps [27].

The MMA of the ACTC promoters and the experimental evidence are shown in Figure 8 (top). While the region proximal to the TSS is not more dense in predicted TFBSs than other regions, most of the aligned elements cluster next to the near TSS. In addition, the alignment agrees well with the functional annotation available in human and mouse, providing novel orthologous sites in chicken and zebrafish:

1. The second SRF binding site is correctly identified in human, mouse and also in zebrafish.

2. A RREB-1 site that overlaps the SP-1 active site is identified in the MMA. RREB-1 and SP-1 are members of the zinc finger protein family with different binding specificities. However, the consensus of both matrices in JASPAR are very similar, being constituted of several occurrences of the motif CCCC [14].

3. A SQUA site that overlaps the third SRF active site is identified in the MMA. SQUA and SRF are both members of the MADS family [14].

4. A novel forth SRF binding site is located immediately upstream of the experimental first one at the four species.

5. The TATA box is correctly detected in human, mouse and zebrafish as well.

No significant conservation among the sequences was, however, detected in the multiple sequence alignment of the four ACTC promoters (see the alignments in the Supplementary Information).

#### Myoglobin gene

The *Myoglobin *gene is a member of the globin superfamily and is expressed in skeletal and cardiac muscles. The encoded protein is an haemoprotein contributing to intracellular oxygen storage and transcellular facilitated diffusion of oxygen [40].

The promoter of the *Myoglobin *gene in human (MB, GenBank: X00371) and in mouse (RefSeq: NM_013593) has been experimentally characterized [39,41]. In the ABS database [13], the entry A0037 includes the known orthologous binding sites in the respective human and mouse promoters (500 nucleotides, the position +501 is the TSS). The human MB promoter is constituted of a CCAC box (+272), a MEF-2 site (+335) with two surrounding E-boxes (+326, +348) and a TATA box (+469). Using the RefSeq gene annotations [28], we have also identified the corresponding orthologous promoters in chicken and zebrafish (RefSeq: NM_203377, NM_200586).

We have then aligned the four promoters and compared the resulting MMA with the functional annotations detailed above. The multiple TF-map alignment of the four orthologous promoters of MB contains several of the functional sites in human and mouse, detecting some of the orthologs in the other two species. The output coverage is again very small.

The MMA of the MB promoters and the experimental evidence are shown in Figure 8 (bottom). Most of the aligned elements are present next to the TSS, while this spatial trend is not observable in the predictions at each promoter. The alignment also contains several of the functional human and mouse sites, providing their counterparts in chicken and zebrafish:

1. A RREB-1 site that overlaps the functional CCAC box is identified in the MMA. In fact, the RREB-1 matrix consensus in JASPAR represents an A/C rich area that contains the CCAC motif [14].

2. The TATA box is correctly detected in the four species.

The multiple sequence alignment of the four MP promoters did not reveal any significant conservation (see the alignments in the Supplementary Information).

#### Even-skipped stripe 2 enhancer

Proximal promoters are adjacent to the gene. Enhancers, however, are other type of regulatory regions (typically 500 – 1,000 nucleotides long) positioned several kilobases upstream or downstream of the regulated gene. Such elements can function in either orientation, being distance and position independent [42]. The regulatory logic of enhancers is different from the promoters, allowing a great plasticity in the arrangement of the TFBSs (e.g. non-collinearity [22]). Enhancers are constituted of multiple binding sites to recruit four or five different TFs that define space and time specific aspects of gene expression [43]).

The body patterning of early embryo in *Drosophila *is governed by a hierarchy of maternal and zygotic genes. In particular, maternal and gap gene factors together control pair rule gene expression in 7 alternating stripes, which in turn regulate segment polarity and homeotic gene expression in 14 stripes [44]. The stripe 2 enhancer of the pair-rule gene *Even-skipped *has been experimentally characterized in several species of *Drosophila*, showing considerable evolutionary change in the binding site composition and spacing [45]. Such annotations have been extensively used to train several computational regulatory module prediction approaches [46-48].

**Figure 9:**
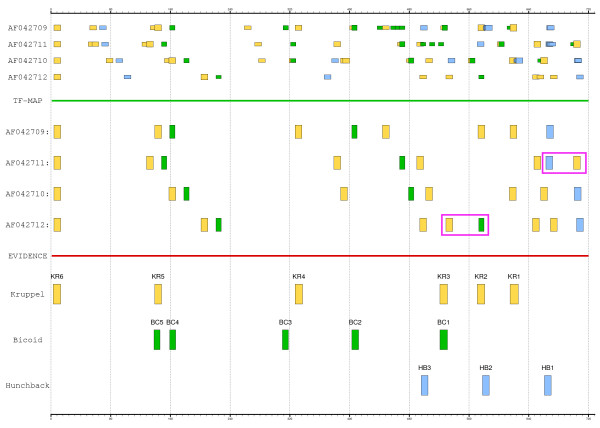
The TRANSFAC predictions of bicoid (in green), hunchback (in blue) and Kruppel (in yellow) zygotic factors on the stripe 2 enhancer of the *Even-skipped *gene in *D. melanogaster*, *D. yakuba*, *D. erecta *and *D. pseudoobscura *(EVE, GenBank: AF042709, AF042710, AF042711 and AF042712), the resulting MMA and the experimental evidence. The TF-map alignment agrees well with the experimental annotation of the enhancer in *D. melanogaster*, as most experimentally annotated TFBSs are successfully covered by the MMAs. Non-collinear rearrangements in the MMA are denoted with a red square (see main text). The graphical representation of the maps and the alignments has been produced with the program gff2ps [27].

The stripe 2 enhancer of the *Even-skipped *gene (EVE, GenBank: AF042709 (*D. melanogaster*), AF042710 (*D. yakuba*), AF042711 (*D. erecta*), AF042712 (*D. pseudoobscura*)) is governed by 17 TFBSs of four TFs [45]: 2 activators (bicoid and hunchback) and 2 repressors (giant and Kruppel). We have obtained from TRANSFAC 8.4 [16] the PWMs to recognize bicoid (I$BCD_01), hunchback (I$HB_01) and Kruppel sites (I$KR_01). We will focus therefore only on the occurrences for these three TFs. The positions of the experimentally verified binding sites in the *Drosophila melanogaster *enhancer are [45]: bicoid (+138, +159, +310, +403, +521), hunchback (+496, +578, +661) and Kruppel (+3, +139, +327, +521, +571, +615). We have obtained the TF-maps of bicoid, hunchback and Kruppel in these enhancers (threshold score 50%). We have then aligned the four enhancer maps allowing non-collinear rearrangement in the alignments (parameter c = 1, see Section Non-collinear TF-map alignments). We have compared the resulting MMA with the available functional annotations. Matches to one of the elements in overlapping sites for activators/repressors are considered to be correct. The MMA of the four orthologous enhancers and the experimental evidence are shown in Figure 9.

Despite we trained our algorithm specifically on human-mouse-chicken-zebrafish genes (vertebrates), the MMA of the four *Drosophila *enhancers still agrees well with the regulatory annotation available for *Drosophila melanogaster*, providing the orthologous known sites in the other species and two additional putative non-collinear rearrangements (see Figure 9):

1. Five out of six known Kruppel sites are correctly identified in the four enhancers.

2. Three out of five known bicoid sites are identified in the four enhancers.

3. Two out of the three hunchback sites are identified.

The MMA contains a non-collinear rearrangement between the HB1 site and the KR1 site in *D. erecta *(see Figure 9). The HB1 site is not detected in *D. erecta *and *D. pseudoobscura *in the conventional sequence alignment of the four enhancers [45]. Non-collinearity is also observed between the BC2 site and a hypothetical Kruppel site predicted in *D. pseudoobscura*.

## Discussion

Among the many codes that shape the sequence of the genomes, the one regulating their transcriptional activity remains remarkably elusive. Indeed, it is usually impossible to infer the specific spatial and temporal expression pattern of a given genomic locus simply from the analysis of the sequences presumably involved in its regulation. It is well known that the initiation of the transcription by RNA Polymerase II requires the interaction between this enzyme and a number of TFs that bind to the DNA sequence in the promoter region upstream of the transcription initiation site. While transcription factors bind short DNA motifs on the promoter region, these motifs are often degenerated, and their effective recognition by the factors is dependent on the structural conformation of the region harboring them.

As a result of these and other circumstances, the relation between primary sequence and regulatory code is far from being trivial. Indeed, recent genome-wide studies based on chromatin immunoprecipitation (ChIP) of DNA bound by promoter-associated proteins, followed by either direct sequencing of the bound region or hybridization in a tiling array (ChIP-chip [49-51]) underlined the complexity of this relationship. Often no occupancy by a given TF has been experimentally detected for many genomic sites where binding motifs can be computationally predicted [52,53]. Therefore, promoter regions of genes sharing similar expression programs often do not show nucleotide sequence conservation.

To overcome this limitation, we introduced recently a novel approach based on abstracting the nucleotide sequence of gene promoters and replacing it by a sequence of labels, each label denoting, at a specific location on the primary sequence, the TF for which a known binding site has been predicted. We used the term TF-maps for denoting such sequences of labels [9]. Pairwise alignments between TF-maps can occasionally reveal underlying configurations of TFBSs shared by co-regulated genes, which escape detection by typical nucleotide sequence comparisons.

Here, we introduce the multiple TF-map alignments. Multiple comparisons increase the power to detect the underlying features common across the compared elements by increasing the signal to noise ratio. Multiple sequence comparisons, in particular, have been used to identify regulatory motifs and coding exons in genomic sequences [54,55]. The rationale is that since the probability of mutation is lower in functional than in non-functional regions, by increasing the number of sequences in the comparisons, nucleotide divergence increases in non-functional regions proportionally more than in functional ones, producing a sharper contrast in the sequence conservation landscape [56,57]. A similar rationale can be applied to the multiple-TF map alignments. TF-maps, obtained usually through computational predictions of binding sites, contain many non-functional elements (i.e. false positive hits). One expects that, among multiple TF-maps corresponding to genes with similar expression patterns, only the functional elements (i.e. the "bona fide" TFBSs) will be conserved.

Indeed, as we have shown, the main effect of the multiple map alignments (MMA) is the dramatic reduction in the number of predicted TFBSs that typically result after a PWM-based search (see Figure 8). For instance, we aligned 157 human sites to 197 mouse sites, 229 chicken sites and 167 zebrafish sites mapped in the respective *Actin α-cardiac *gene promoter orthologs. The resulting multiple TF-map alignment included only 14 TFBSs, which approximately represents a 13-fold reduction. In addition, most aligned sites in the MMAs are concentrated in the proximal promoter region of each gene (200 nucleotides upstream of the TSS). This gain in specificity is not simply due to the selection of an arbitrary set of non-overlapping TFBSs, since many experimentally annotated TFBSs on these promoters are successfully covered by the resulting MMAs.

We have trained our approach on a human-mouse-chicken-zebrafish dataset mostly constituted of tissue-specific genes, because of the enrichment of such a promoter class in the regulatory annotation literature [36,39]. A recent study states, however, that the classical TATA-box promoter architecture of such genes represents a minority of the set of mammalian promoters in human and mouse [58]. The structure of TATA-independent promoters occurring within a CpG island is more flexible and evolvable. We consider, however, the evaluation of the MMA presented here is still correct as our approach does not distinguish the TATA-box and the other core promoter elements from the rest of TFs during the alignment. MMAs can therefore also deal with flexible regulatory rearrangements (see the TF-map characterization of the *Even-skipped *stripe 2 enhancer). In addition, in a previous study the TF-map alignments showed to be also effective in more general regulatory datasets that contained both classes of promoters [9]. Map alignments were introduced in the early 1980s to compare restriction enzyme maps [59]. Several improvements on the basic pairwise algorithm were developed since then [60] but this is the first time a multiple alignment implementation is proposed. In practice, guaranteeing the optimal solution to the multiple sequence comparison problem is difficult. Our approach is based on the progressive alignment paradigm, which produces not necessarily optimal alignments despite the results are biologically meaningful [4]. Here we have generalized the data structures and algorithms shown in a previous work for the pairwise comparison [9], to deal with multiple maps without adding supplementary complexity. Thus, the cost of the final implementation is proportional to the number of pairwise comparisons performed by the progressive approach.

In addition, we have redefined the way in which the dynamic programming matrix is processed in order to capture non-collinear configurations in the maps of regulatory elements. We have shown an example in which non-collinearity helps to find rearrangements of TFBSs that can not be detected using a conventional linear approach. Despite it is actually very difficult to train the non-collinear algorithm due to the lack of abundant experimental annotations, we believe this kind of approach will be very important in the future as collinearity can not be always assumed in regulatory regions [12].

**Figure 10:**
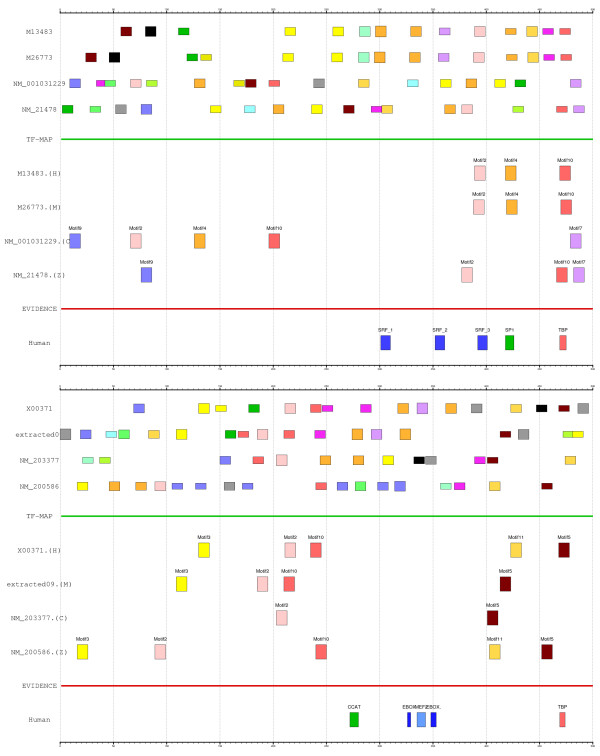
On top, the MEME novel patterns discovered on the human-mouse-chicken-zebrafish promoters of the *Actin α-cardiac *gene, the resulting MMA and the experimental evidence. MEME motifs in the alignment supported by real sites: (Motif2, GACCAAATAAGGCAA, SRF), (Motif4, GGCAGGGGAGAGGAT, SP1), (Motif10, TATAAAG, TBP). At bottom, the MEME patterns on the human-mouse-chicken-zebrafish promoters of the *Myoglobin *gene, the resulting MMA and the experimental evidence. MEME motifs in the alignment supported by real sites: (Motif5, TATAAAA, TBP). The motifs are displayed as boxes along the promoter. MEME motifs were obtained with these parameters: -nmotifs 20 -minw 6 -maxw 15. Both graphical representations have been produced with the program gff2ps [27].

The TF-map alignments are able to unveil characteristic regulatory patterns that are difficult to be detected at the sequence level. To test this hypothesis, we have used collections of position weight matrices as external mapping functions. However, the TF-map alignment algorithm can also deal with other kind of regulatory maps as those produced by pattern discovery programs. For instance, we have used the MEME program [17] to discover novel motifs (number of motifs: 20, minimum site length: 6, maximum site length:15) in the promoters of the *Actin α-cardiac *and *Myoglobin *genes. We have then performed the MMA of such patterns. The input motifs and the resulting alignment in both cases are shown in Figure 10. Such an approach can be useful to enrich the results obtained when the same promoters are aligned using JASPAR (see Figure 8).

While here we have focused on TF-maps, the algorithms and software that we have developed can also be applied, in principle, to any other problem in which the primary sequence can be annotated with higher-order features (that is, it can be mapped into a sequence of labels denoting these features). Thus, comparisons between the annotations (instead of primary sequences) can reveal more biological clues. Examples could include comparisons between exons/introns that have been annotated with matches to binding motifs for splicing regulatory factors [61]. The MMAs, in this case, could reveal classes of exons whose splicing is regulated in a similar way. Or comparisons between protein sequences which have been annotated with functional (PFAM, [62]) or structural domains. The MMAs could help here to infer functional super-families. Or comparisons between entire genomes, which have been annotated with the biological functions (for instance, using Gene Ontology (GO) terms [63] of the genes across the genomes). The MMAs could help to investigate whether function and chromosomal localization are related.

## Conclusion

In general, as the functionality of the primary sequence becomes better understood, more innovative alignment techniques between higher-order representations of the sequences, such as the approach we presented here, will become increasingly useful to uncover the features that underlie common functionality.

## Authors' contributions

EB, RG and XM conceived and designed the experiments. EB performed the experiments. EB, RG and XM analyzed the data. RG contributed reagents/material/analysis tools. EB, RG and XM wrote the paper.
